# SP1 and p23 play a crucial role in the circadian target gene induction of activated aryl hydrocarbon receptor in human breast cells

**DOI:** 10.1007/s10565-025-10080-0

**Published:** 2025-09-12

**Authors:** Melina Mihelakis, Tanina Flore, Gilbert Schönfelder, Michael Oelgeschläger, Norman Ertych

**Affiliations:** 1 German Centre for the Protection of Laboratory Animals (Bf3R), German Federal Institute for Risk Assessment, Diedersdorfer Weg 1, 12277 Berlin, Germany; 2 Department of Clinical Pharmacology and Toxicology, Charité - Universitätsmedizin Berlin, Berlin, Germany

**Keywords:** Circadian, AHR, BMAL1, CLOCK, P23, SP1, Xenobiotic

## Abstract

The Aryl Hydrocarbon Receptor (AHR) is a crucial mediator of cellular responses upon exposure to environmental pollutants. Initially described as central activator in xenobiotic metabolism, recent research has unveiled additional layers of complexity in AHR function and regulation. The circadian rhythm is a fundamental regulatory process that modulates various physiological processes, including AHR activity. Our recent findings show that AHR-dependent gene induction is subject to circadian rhythmicity. While some studies suggest a circadian AHR gene transcription in various tissues, a comprehensive mechanistic understanding of the circadian AHR regulation remains elusive. This mechanistic study aimed to elucidate the circadian regulation of AHR target gene induction upon dioxin treatment in human breast cells. To acquire a more profound understanding of the intricacies of AHR regulation, we conducted a systematic analysis of the molecular co-factors and their interactions in circadian synchronized cells. Our results show circadian regulation of AHR transcriptional activity at the CYP1A1 promoter upon dioxin treatment. This appears to be orchestrated by the core clock components BMAL1/CLOCK, which directly interact with AHR in circadian synchronized cells. Additionally, we identified SP1 as an important positive and p23 as an essential negative regulator of circadian AHR activity. The understanding of these interactions is crucial for elucidating the molecular relationship between the circadian clock and cellular responses to environmental stimuli. Such knowledge is of vital importance for the application of New Approach Methods (NAMs) as part of a weight-of-evidence (WoE) approach in the next generation of risk assessments.

**Graphical Abstract:**

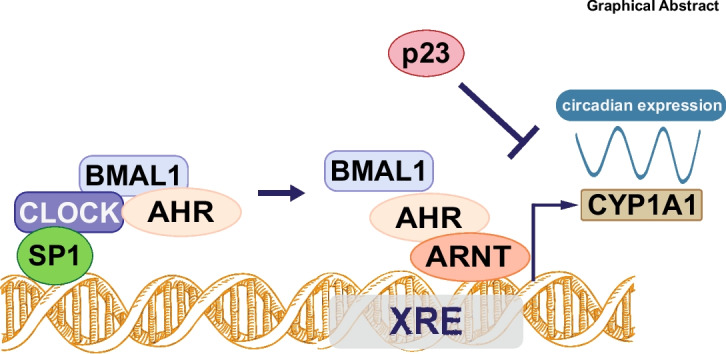

**Supplementary Information:**

The online version contains supplementary material available at 10.1007/s10565-025-10080-0.

## Introduction

The Aryl Hydrocarbon Receptor (AHR) is a ligand-activated transcription factor that plays a pivotal role in mediating cellular responses to both endogenous and exogenous substances (Barouki et al. 2012). As part of the first line of cellular defence, the AHR was originally identified as an initiator of xenobiotic metabolism. Therefore, understanding its function and regulation is of utmost importance for assessing health risks and disease development. Meanwhile, the AHR has been identified as a versatile regulator involved in a wide range of biological processes (Larigot et al. 2022; Opitz et al. 2023). This underscores the importance and complexity of understanding its function and regulation.

The AHR is a member of the bHLH-PAS protein family (basic Helix-Loop-Helix - Period/ARNT/Single minded) and contains three important domains: The bHLH domain, two PAS domains and a C-terminal region (Larigot et al. 2018). The bHLH and PAS domains enable the dimerization with its central co-factor ARNT upon ligand binding and the subsequent DNA binding (Dai et al. 2022). These domains also facilitate interaction with chaperones such as Hepatitis B virus X-associated protein 2 (XAP2), Heat Shock Protein 90 (HSP90) or the co-chaperone p23, independent of a ligand (Kazlauskas et al. 2001), and mediate the nuclear import and export of AHR. In the nucleus, AHR interacts with co-activators and co-repressors such as SP1, p300-CBP or SRC-1, via its three C-terminal subdomains (Kobayashi et al. 1996, 1997; Taylor et al. 2009).

In the absence of external ligands, AHR is mainly located in the cytoplasm and forms a stable cytosolic complex with HSP90, XAP2, and p23 (Larigot et al. 2018; Wen et al. 2023). Here, HSP90 sequesters AHR, which enables AHR to bind to various ligands that enter the cell (Soshilov and Denison 2011). The co-chaperone p23 stabilizes the AHR-HSP90 complex and reinforces the sequestration by masking the nuclear translocation signal in the bHLH domain of the AHR protein (Cox and Miller 2004). In addition, the cytosolic co-factor XAP2 facilitates proper folding of AHR in the cytoplasm, which then improves AHR signalling (Fujii-Kuriyama and Kawajiri 2010).

In the presence of ligands, such as polycyclic aromatic hydrocarbons or dioxins are present, the cytosolic co-factors dissociate and AHR translocate into the nucleus (Denison et al. 2002; Larigot et al. 2018). There it forms a nuclear complex with its central co-factor ARNT and bind together with multiple transcriptional co-activators and co-repressors such as SP1, p300-CBP or SRC-1 to the dioxin response elements (DREs) or xenobiotic response elements (XREs) within the genome (Kobayashi et al. 1996, 1997; Taylor et al. 2009). Subsequently, activated AHR/ARNT complex induces the expression of target genes, including members of the like phase I metabolism enzyme *Cytochromes P450* family (e.g. *CYP1A1*) and phase II metabolism enzyme like the *UDP glucuronosyltransferase 1* family (e.g. *UGT1A6)* or *Aldehyde Dehydrogenase* family members (e.g. *ALDH3A1*) *(*Granados et al. 2022*)*. Additionally, the *AHR repressor* (*AHRR*) is an AHR target gene that acts as an antagonist to the AHR/ARNT/XRE complex and supresses AHR activity (Mimura et al. 1999). Together with the proteasomal degradation of AHR, the AHRR serves as a repressor of this pathway and prevents the overstimulation by excess ligands in a negative feedback loop (Haarmann-Stemmann and Abel 2006).

Interestingly, the strength of AHR-dependent transcription is subject to the circadian rhythm, indicating that the entire process is also dependent on the time of day (Shimba and Watabe 2009; Jaeger et al. 2017; Ndikung et al. 2020; Tischkau 2020). Recent studies demonstrated a rhythmic basal expression of *CYP1A1*, but more relevant, the induction level upon xenobiotic exposure seems to be highly regulated in a circadian manner (Huang et al. 2002; Qu et al. 2010; Schmitt et al. 2017; Ndikung et al. 2020). The circadian regulation of the AHR is an emerging field of research that aims to elucidate the molecular interplay between the circadian clock and cellular responses to environmental triggers.

At the molecular level, the circadian clock is a self-sustaining transcriptional-translational feedback loop that adjusts the activity of core clock genes such as *CRY1/2*, *BMAL1*, *PER1/2/3*, *ROR*, *CLOCK* and *RevErbα* among others in a 24-hour cycle (Buhr and Takahashi 2013; Partch et al. 2014). These transcription factors subsequently regulate physiological processes, such as blood pressure, body temperature and metabolic activity (Patke et al. 2020). In detail, BMAL1 and CLOCK form a heterodimer that initiates the expression of their own negative regulators PER1/2/3 and CRY1/2. Subsequently, a multimeric complex containing PER and CRY translocate in the nucleus and represses time-delayed the transcriptional activity of the BMAL1/CLOCK complex (Partch et al. 2014; Takahashi 2017). In addition, BMAL1/CLOCK promotes the transcription of *RevErbα*, which in turn represses BMAL1 expression (Preitner et al. 2002). This core clock machinery drives the expression of so-called clock-controlled genes, which subsequently regulate a wide range of physiological processes, including xenobiotic metabolism (Patke et al. 2020; Ayyar and Sukumaran 2021).

Interestingly, the clock genes BMAL1, PER and CLOCK share the same PAS domains as AHR and ARNT (Shimba and Watabe 2009). It is not surprising that first studies reported a direct or indirect link of the circadian rhythm and the AHR signaling pathway (Tischkau 2020). This structural similarity suggests a potential interaction between circadian clock genes and the AHR (Wang et al. 2014). Indeed, a recent study reported that BMAL1 interacts with murine AHR in mouse ovaries, but their direct interaction has not been shown in human tissues or cells yet (Jaeger et al. 2017; Tischkau 2020). Besides these observations, several animal studies have demonstrated that the expression of *Ahr* itself exhibits circadian rhythmicity in certain tissues. For instance, *Ahr* expression in the liver, lungs, and thymus of rodents shows a circadian pattern (Richardson et al. 1998; Huang et al. 2002; Tischkau 2020). However, the circadian regulation of *AHR* appears specific to certain tissues and species, as rhythmic gene expression in human tissues or cells is rarely reported. The circadian regulation of AHR appears to be a complex and dynamic process. This has a significant impact on the understanding of diseases and pharmacokinetics, as well as toxicological assessments. (Mihelakis et al. 2022).

The present study demonstrates that the AHR/ARNT complex interacts with the clock core component BMAL1/CLOCK in synchronised cells. Subsequently, the transcriptional activity of the AHR/ARNT complex follows a circadian rhythm. Moreover, this circadian regulation is influenced by two AHR co-factors with opposing activities. SP1 was identified as a positive regulator that is under circadian regulation. In contrast, the chaperone p23 functions as a negative regulator specifically in circadian-synchronized cells Our findings contribute to unraveling the complexity of AHR signaling and offer new insights into the biological roles and regulatory mechanisms of AHR. This will expand the understanding of the role of AHR in physiological homeostasis and pathogenesis.

## Methods

### Cell Lines and Culture

The human breast epithelial cell line *hTERT*-HME1 (ATCC) was grown in mammary epithelial growth medium (MEGM, Lonza) supplemented with the mammary epithelial cell growth kit (MEM, ATCC) and penicillin/streptomycin (PAN-Biotech). Hydrocortisone (ATCC) with the defined concentration of 0.1 µg/mL was added separately. The breast epithelial cell line M13SV1 was cultured in Michigan State University-1 medium (Kao et al. 1995) supplemented with penicillin/streptomycin (PAN-Biotech) and 10 % fetal bovine serum (Sigma). The generation of HME1 luciferase-reporter cell lines (*hTERT*-HME1-PLB) was previously described (Ndikung et al. 2020). All cell lines were grown in a humidified atmosphere at 37 °C and 5 % CO_2_.

### Synchronization and Treatment Procedures

To synchronize the cells, they were seeded at a density of 100% confluency and, after 24 h, they were entrained with 1 µM dexamethasone for 1 h. Upon two washing steps with DPBS, the cells were treated with the respective substances dissolved in MEGM or MSU-1 for 12, 24, 36 or 48 h. Cells serving as non-synchronized control were seeded at a density of 30- 40% to avoid confluency and self-synchronization. After 24 h, the non-synchronized cells were equally treated with the respective substances as the synchronized cells. hTERT-HME1 and M13SV1 cells were seeded at a density of 1 x10^6^ and 0.3 x 10^6^ cells/well for a 100% and 30% confluency in a 6-well plate, respectively.

### siRNA transient transfection

M13SV1 cells were seeded to have a 70% confluency on the day of transfection. 120 µM of following siRNAs were transfected using the transfection reagent HiPerFect^TM^ (Qiagen): 5’-GGAUGUAGAUUUACCAGAA-3’ for *p23*, 5´-GCCAAUAGCUACUCAACUA-3´ for *SP1*, 5´- AAGCGGCAUAGAGACCGACUU-3´ for *AHR*, 5´-GCAGAUAUCUCUAUGAUUG-3´ for HSP90, 5´-CCAGUUCCUCUGUGACAUCAA-3´ for *XAP2*, 5´-GCAACGAUCGUGGACUAUC-3´ for *AHRR*, and 5´-UGGGCUCAAGGAGAUCGUUUA-3´ for *ARNT*. siRNA was mixed with medium without supplements or antibiotics to a final volume of 100 µL. HiPerFect^TM^ (12 µL) was added and immediately mixed using a vortex mixer for 10 sec. The transfection solution was incubated for 10 min at RT and in the meantime the medium of the cells in the plate was exchanged for medium without supplements and antibiotics (1 mL). The transfection mix was added dropwise to the cells and incubated for 5-6 h. After the incubation, the cells were washed and supplemented with growth medium as described. 48 h after transfection, the cells were further analyzed by Western blotting and RT-qPCR.

### Plasmid DNA transfection

For DNA transfection 1.6 x 10^6^ M13SV1 cells were resuspended in 400 µL MSU-1 complete medium, transferred to a 4 mm electroporation cuvette (Peqlab, 71-2030) containing 15 µg plasmid DNA and electroporated using the Gene Pulser Xcell System (Biorad) with 950 µF, 220 V, ∞ Ω. The electroporated cells were transferred to a well of a 6-well plate and further analyzed 48 h after transfection by Western blotting or RT-qPCR.

HME1-PLB cells were transfected at 70% confluency with 3 µg plasmid DNA and 10 µL Torpedo^DNA^ transfection reagent (#60612, ibidi) according to the manufacturer’s protocol. 48 h after transfection, the cells were further analyzed by circadian reporter bioluminescence assay. Cells were transfected with pcDNA3 (V79020, Invitrogen), pGFP-N2-p23, a gift from William Chan (Pappas et al. 2018) and CMV-SP1, a gift from Robert Tjian (#12097, Addgene).

### Quantitative Real-Time PCR

Total RNA was isolated according to the manufacturer’s protocol (RNeasy Mini Kit; Qiagen) including a DNase digestion step, and concentration was determined using a NanoDrop^TM^ spectrophotometer (ThermoFisher Scientific). The cDNA synthesis was performed with 1 µg RNA applying the High-Capacity cDNA reverse transcription kit (Applied Biosystems). RT-qPCR was performed on a Quantstudio 7 FLEX System (Applied Biosystems). 5 ng of each cDNA sample was set up in triplicates using Power Up SYBR Green Mix (Applied Biosystems) with the respective primers: 5’-CTCCGTGGCCTTAGCTGTG-3’ and 5’-TTTGGAGTACGCTGGATAGCC-3’ for *B2M*; 5’-TTTGAGAAGGGCCACATCCG-3’ and 5’-AGGCCTCCATATAGGGCAGAT-3’ for *CYP1A1*; 5’-AACGGAGGCCAGGATAACTG-3’ and 5’-GACATCAGACTGCTGAAACCCT-3’ for *AHR;* 5’-AGCGGAGATGAAAATGAGGA-3’ and 5’-AGTTCCGATTCGCACAGACT-3’ for *AHRR*; 5’-GATTCCAAGCATAAAAGAACGGAC-3’ and 5’-GAATCATCTTCCCAGTCTTTCCAA-3’ for *P23*; 5’-TTGAAAAAGGAGTTGGTGGC-3’ and 5’-TGCTGGTTCTGTAAGTTGGG-3’ for *SP1*; 5’-CAAGCTAACCATCTTACGCATGGCAG-3’ and 5’-TGGACCACCACGAAGTGAGGTTC-3’ for *ARNT*; 5’-GAGCTCATCATTGGCAAGAAGTT-3’ and 5’-TACAGGACCACATGCTGGATGTC-3’ for *XAP2.* The mRNA expression of each gene of interest was normalized to the endogenous *B2M.*

### Cell fractionation and de novo CYP1A1 expression

The *de novo* synthesis of mRNA was determined via RT-qPCR employing primers that recognized the intron regions of the RNA before it was spliced and modified into mRNA. Since *de novo* RNA is predominantly found in the nucleus, a cell fractionation procedure was conducted prior to the RNA purification process. The cells from a well of a 6-well plate were harvest and lysed in 100 µL lysis buffer (150 mM NaCl, 10 mM Tris HCl [pH 7.4], 0.15 % Igepal ® CA-630 (Sigma-Aldrich)) on ice for 5 min and layered carefully on 250 µL sucrose buffer premixed tubes (150 mM NaCl, 10 mM Tris [pH 7.4], 0.25 g/mL sucrose), emerging two phases. After centrifugation (3,500 x g/10 min), the supernatant (cytosolic fraction) was discarded, and the pellet (nucleolar fraction) was washed briefly with ice-cold DPBS. The nucleolar RNA was isolated by adding 350 µL RLT buffer to the pellet and by following the protocol from the *RNeasy Mini Kit* ® (Qiagen) including a DNase digestion step. After measuring the RNA on a NanoDrop^TM^ spectrophotometer (ThermoFisher Scientific), the cDNA synthesis was performed with 350 ng RNA, applying the High-Capacity cDNA reverse transcription kit (Applied Biosystems). RT-qPCR was performed on a Quantstudio 7 FLEX System (Applied Biosystems). Therefore, 175 ng of each cDNA sample was set up in triplicates using Power Up SYBR Green Mix (Applied Biosystems) and the primers 5´-TACCCTGTCCAGGGTTTGACA-3´ and 5´-AATCACTGTGTCTGCAGAACAC-3, which specifically identified the *de novo CYP1A1* expression, were applied.

### Circadian Reporter Bioluminescence Assay

Cells were seeded into a white 96-well plate (Greiner) at a density of 1x10^5^ cells/well and after 24 h, entrained with 1 µM dexamethasone for 1 h, washed twice with PBS and cultivated in MEGM/0.5 mM D-Luciferin (PJK Biotech). Non-synchronized (control) cells were seeded at a density of 0.2x10^5^ cells/well. The bioluminescence reporter signal was measured every 30 min for 72 h with a microplate reader (Synergy Neo2 Biotek^®^) applying a photon count of 6.5 sec/well and an internal gain of 125. The obtained signals were analyzed by the ‘ChronAlyzer’ software (Ndikung et al. 2020).

### Western Blotting

Cells were seeded and treated as described above. After 12, 24, 36 or 48 h of treatment, cells were harvested and lysed in 100 µL lysis buffer (50 mM Tris/HCl [pH 7.4], 150 mM NaCl, 5 nM EDTA [pH 8.0], 5 nM EGTA, 1% NP-40, 0.1% Na-Desoxycholat, 0.1% SDS) supplemented with protease (cOmplete^TM^, Roche) and phosphatase (PhosSTOP^TM^, Roche) inhibitors. 50 µg of protein solution was separated with SDS-PAGE and transferred to a nitrocellulose membrane (Bio-Rad). The membrane was blocked in Intercept (TBS) Blocking Buffer (LI-COR) and subsequently incubated with primary antibodies over night at 4 °C on a rocky shaker. Next, the membrane was washed and incubated with secondary antibodies for 1 h at RT. The following primary antibodies diluted in 3% w/v BSA/TBST were used: AHR antibody (1:1000; Abcam; ab190797), ARNT antibody (1:1000; Cell Signaling; #3414), p23 antibody (1:10000; Invitrogen; #MA3-414), HSP90 antibody (1:1000; Cell Signaling; #4877), XAP2 antibody (1:1000; Novus Biologicals; NB100-127), SP1 antibody (1:2000; Novus Biologicals; NB600-233), CLOCK antibody (1:1000; Cell Signaling; #5157), BMAL1 antibody (1:1000; Novus Biologicals; NB100-2288), BMAL1 antibody (1:500; Santa Cruz; sc-365645), β-Actin antibody (1:5000, Abcam, ab8227) and GAPDH antibody (1:1000, Cell signaling; #5174). As secondary antibodies diluted in 3% w/v BSA/TBST, goat-anti-mouse IgG (H+L) (DyLight ™ 680 Conjugate, Cell Signaling, #5470) and goat-anti-rabbit IgG (H+L) (DyLight™ 800 4X PEG Conjugate, cell signaling, #5151) were used. Detection was conducted with an Odyssey CLx Imaging System (Odyssey CLx, LI-COR) and the blots were analyzed using the Image Studio™ Lite Software (LI-COR).

### Immunoprecipitation

Cells were seeded and treated accordingly. The harvested cells were lysed in ice-cold immunoprecipitation lysis buffer (150 mM Tris HCl, [pH 7.5], 150 mM NaCl, 0.25% NP-40, 10% glycerol, 0.1 mM EDTA [pH 7.5], 1 mM DTT), supplemented with protease (cOmplete^TM^, Roche) and phosphatase (PhosSTOP^TM^, Roche) inhibitors and sonicated (4-5 times, 20 cycles, 0.5 kHz, 80% amplitude, on ice). The cell debris was removed by centrifugation at 4 °C, for 15 min at 20,000 x g. As input control, 50 µg protein samples were prepared for SDS-PAGE and 1 mg lysates with a concentration of 7 mg/mL were prepared for immunoprecipitation. The samples were incubated in rotation at 4 °C for 1.5 h with 1.5 µg of the following antibodies: AHR antibody (abcam, ab190797) or BMAL1 antibody (Novus Biologicals; NB100-2288). The immunocomplexes were precipitated using equilibrated 50% slurry protein G Sepharose beads (rotation at 4 °C for 1.5 h). After three gentle washing steps, the samples were boiled at 95 °C for 2 min in Laemmli sample buffer. Input and immunoprecipitants were analyzed by SDS-PAGE and Western blotting.

### Statistical Analysis

Data analysis and preparation of graphs were conducted with Microsoft Excel or GraphPad Prism 9. Statistical significance was determined using an unpaired, one-tailed moderated Student's *t*‐test. Statistical significance was accepted at *P* ≤ 0.05 and the *P*-values as well as non-significance (*P*≥ 0.05), denoted with “n.s.”, are indicated in the figures.

## Results

### Circadian regulation of the CYP1A1 promoter in vitro

**Fig. 1:**
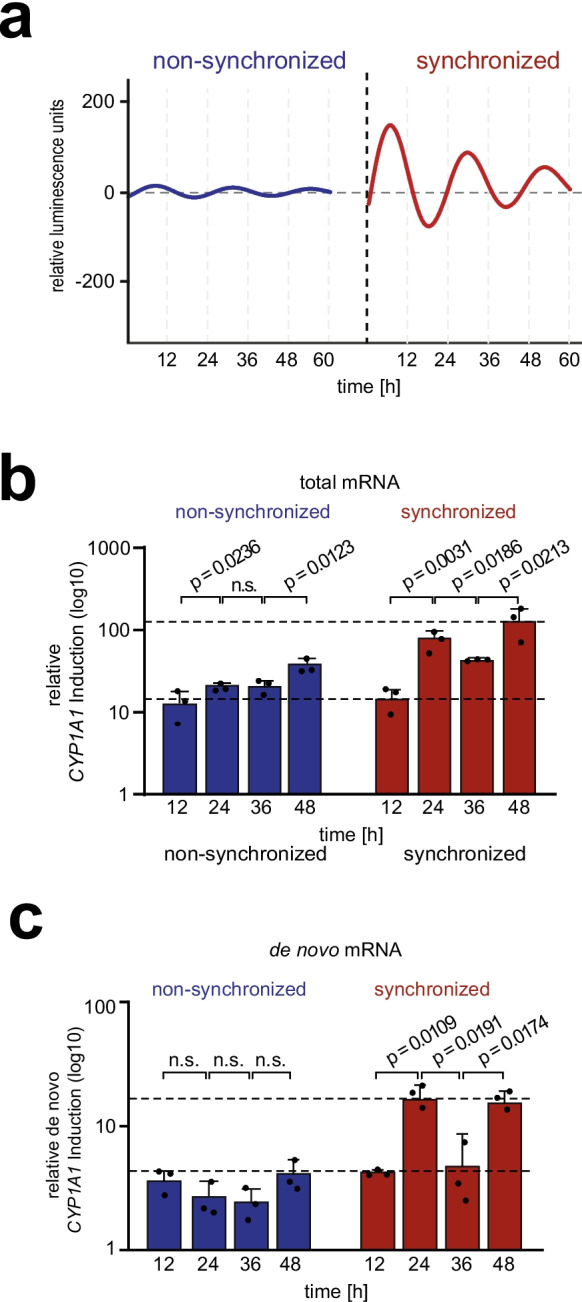
*CYP1A1 induction upon TCDD exposure is regulated by circadian promoter activation*. **a**) Representative curves of fitted bioluminescence measurements of non-synchronized (blue) and synchronized (red) circadian HME1 reporter cells, expressing *LUCIFERASE* under control of the human *PER2* promoter. For the synchronization confluent cells were treated with 1 µM dexamethasone for 1 h. For the non-synchronized condition, the cells were cultured at a semi-confluent stage and left untreated. The cells were monitored for 60 h and the bioluminescence signal was recorded every 30 min and analyzed with the *ChronAlyzer* software. The non-synchronized cells display no rhythmic bio-luminescence signal, whereas synchronized cells show a circadian oscillation with a period of approximal 24 h. **b)** *CYP1A1* induction at different time points in synchronized and non-synchronized HME1 cells upon treatment with 0.5 nM TCDD. At indicated timepoints, *CYP1A1* mRNA after TCDD exposure was analyzed by RT-qPCR. Fold change was calculated based on the DMSO control of the respective timepoint. In synchronized cells, the TCDD-mediated *CYP1A1* induction displays a circadian pattern peaking at 24 and 48 h. Each bar represents the mean ± SD of three independent experiments. The individual data points from the single experiments are displayed. Statistical significance was determined using an unpaired, one-tailed t-test. **c)** *De novo CYP1A1* induction at different timepoints in non-synchronized and synchronized HME1 cells upon treatment with 0.5 nM TCDD. At indicated timepoints, mRNA was extracted from the nucleus and analyzed by RT-qPCR. The fold change of the *de novo CYP1A1* mRNA after TCDD exposure was calculated based on the DMSO control at the respective timepoint. The TCDD-mediated *de novo CYP1A1* expression exhibits a circadian pattern only in the synchronized cells indicating that the promoter is circadian regulated. Each data point represents the mean ± SD of three independent experiments. The individual data points from the single experiments are displayed. Statistical significance was determined by using an unpaired, one-tailed t-test

As demonstrated in the work of Ndikung et al. (2020), the *CYP1A1* induction mediated by TCDD (2,3,7,8-Tetrachlorodibenzo-p-dioxin) exhibits a circadian oscillation. In this study, the model system and the circadian synchronization process have been characterized in depth. The objective of the present study is to elucidate the mechanism behind this observation. Consequently, the initial focus was directed towards the AHR-mediated promoter activity of *CYP1A1*. To this end, we determined the *CYP1A1* induction upon treatment with TCDD over the course of 48 h in synchronized *hTERT*-HME1-*PER2* (abbreviated as HME1) circadian reporter cells. This reporter cell system allows the visualization of the circadian rhythm by monitoring the oscillating *LUCIFERASE* expression, which is under control of the human *PER2* promoter. First, *hTERT*-HME1-*PER2* cells were treated with 1 µM dexamethasone for 1 h to synchronize all cells in the same circadian phase, or were left untreated for non-synchronized control. Luciferase activity was monitored for 60 h to ensure a stable synchronization over 48 h. To analyze the circadian rhythmicity, the data obtained were analyzed using the ChronAlyzer software (Ndikung et al. 2020), which normalizes the bioluminescence counts (Fig. S1a) and fits the series of measurements to an oscillatory curve. (Fig. 1a). As expected, the HME1 cells exhibited a circadian rhythm with a 24 h period for at least 2 days and thus were suitable for *CYP1A1* induction assays.

To study *CYP1A1* induction, non-synchronized and synchronized HME1 cells were treated with 0.5 nM TCDD for 12, 24, 36 and 48 h, and mRNA levels were determined by RT-pPCR. Total *CYP1A1* mRNA levels in non-synchronized cells did not show a circadian pattern, whereas in synchronized cells a typical circadian expression pattern was observed, with peaks at 24 h and 48 h, respectively (Fig. 1b, S1b). Notably, the mature mRNA showed a slight accumulation after 36 h, which partially masks circadian-driven reduction of *CYP1A1* mRNA in synchronized cells. This finding might be attributable to an altered long half-life of *CYP1A1* mRNA in breast cells, as was previously described when comparing liver and breast cells. (Lekas et al. 2000; Lo et al. 2017). To investigate whether this circadian expression pattern is due to the circadian activity of the *CYP1A1* promoter, we analyzed the *de novo CYP1A1* mRNA levels extracted from the nucleus. The determination of the *de novo CYP1A1* mRNA levels in synchronized cells treated with TCDD showed a significant circadian pattern over time with clear induction peaks at 24/48 h, as well as Low induction at the level of non-synchronized cells at 12/36 h (Fig. 1c, S1c). These findings suggest that the transcriptional activity itself is subject to rhythmic regulation, thereby indicating that AHR-dependent regulation of *CYP1A1* expression is under circadian control.

### Circadian expression of the AHR co-activator SP1

**Fig. 2:**
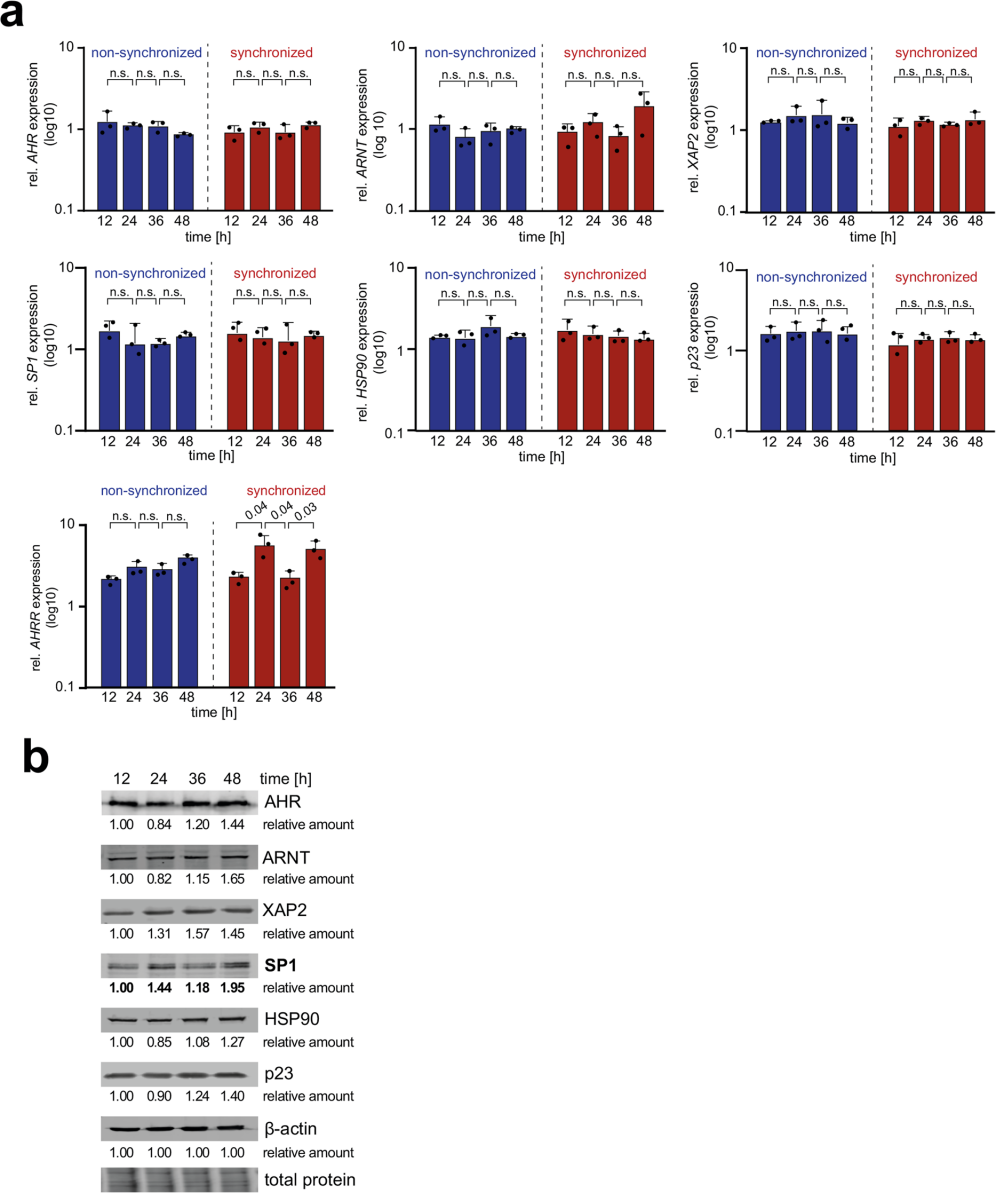
Circadian pattern in protein levels of the AHR cofactor SP1. **a**) To identify AHR co-factors with a circadian expression level, the mRNA fold change of non-synchronized and synchronized HME1 cells after a treatment with 0.5 nM TCDD was analyzed at the indicated timepoints after synchronization. The mRNA levels of *AHR*, *ARNT*, *XAP2*, *SP1*, *HSP90*, *P23* and *AHRR* were analyzed by RT-qPCR. For each timepoint the fold change of the *CYP1A1* expression was calculated based on the DMSO control of the respective timepoint. The TCDD mediated induction of *AHR*, *ARNT*, *XAP2*, *SP1*, *HSP90*, and *P23* remained stable over time in both non-synchronized and synchronized cells. The induction of the AHR target gene *AHRR* displayed under synchronized conditions a circadian pattern with peaking after 24 h and 48 h. Each bar represents the mean ± SD of three independent experiments. The individual data points from the single experiments are displayed. **b)** Representative Western blots from three independent experiments of the AHR co-factors AHR, ARNT, XAP2, SP1, HSP90 and p23 from HME1 cells after 12, 24, 36, and 48 h of synchronization. The protein levels of SP1 show a circadian pattern with peaking at 24 h and 48 h upon synchronization indicating that SP1 protein levels under circadian regulation. β-Actin and total protein stain served as loading control. The relative amounts of proteins were derived from the representative Western blot signal intensity and normalized to β-Actin. The quantification was conducted with Image Studio™ Lite Software (LI-COR Biosciences)

Earlier studies in mice report a circadian expression of *Ahr* in several tissues, whereas in human breast cells the *AHR* expression seems not to be under circadian regulation (Richardson et al. 1998; Huang et al. 2002; Ndikung et al. 2020). To ascertain the extent to which the AHR pathway is subject to circadian regulation, HME1 cells were synchronized or left non-synchronized. The mRNA levels of *AHR*, as well as its co-factors *HSP90*, *XAP2*, and *p23*, and *ARNT* and the transcription factor *SP1*, along with its negative regulator *AHRR*, were determined over time (12, 24, 36, and 48 hours) in the presence of 0.5 nM TCDD. The non-synchronized cells displayed a constant expression level of all analyzed genes over 48 h (Fig. 2a, S2a; blue bars). Interestingly, we also found no circadian expression pattern for the common AHR co-factors in synchronized cells (Fig. 2a, S2a; red bars). However, with the exception of AHRR, a circadian expression pattern was observed, with Lower induction after TCDD exposure at 12/36 h and a peak induction at 24/48 h (see Fig. 2a).

Since the mRNA levels of AHR co-factors are not circadian regulated, we examined the protein levels in synchronized cells of the previously analyzed co-factors by Western blotting. Therefore, we synchronized the HME1 cells and determined the protein levels at 12, 24, 36 and 48 h after synchronization (Fig. 2b). In addition, we quantified the relative signal intensity of each band to support the visual impression of the Western blots. Although, the mRNA levels of the AHR co-factors were not regulated in a circadian manner, we observed a circadian pattern of the protein levels of SP1, with a Low level at 12/36 h and a peak expression level at 24/48 h (Fig. 2b). The derived relative protein levels support this finding, as only SP1 protein exhibited a clear circadian pattern.

Taken together, we found no circadian expression of AHR co-factors at the mRNA level, but SP1 protein levels appeared to be circadian regulated.

### Modulators of circadian associated AHR activity

**Fig. 3:**
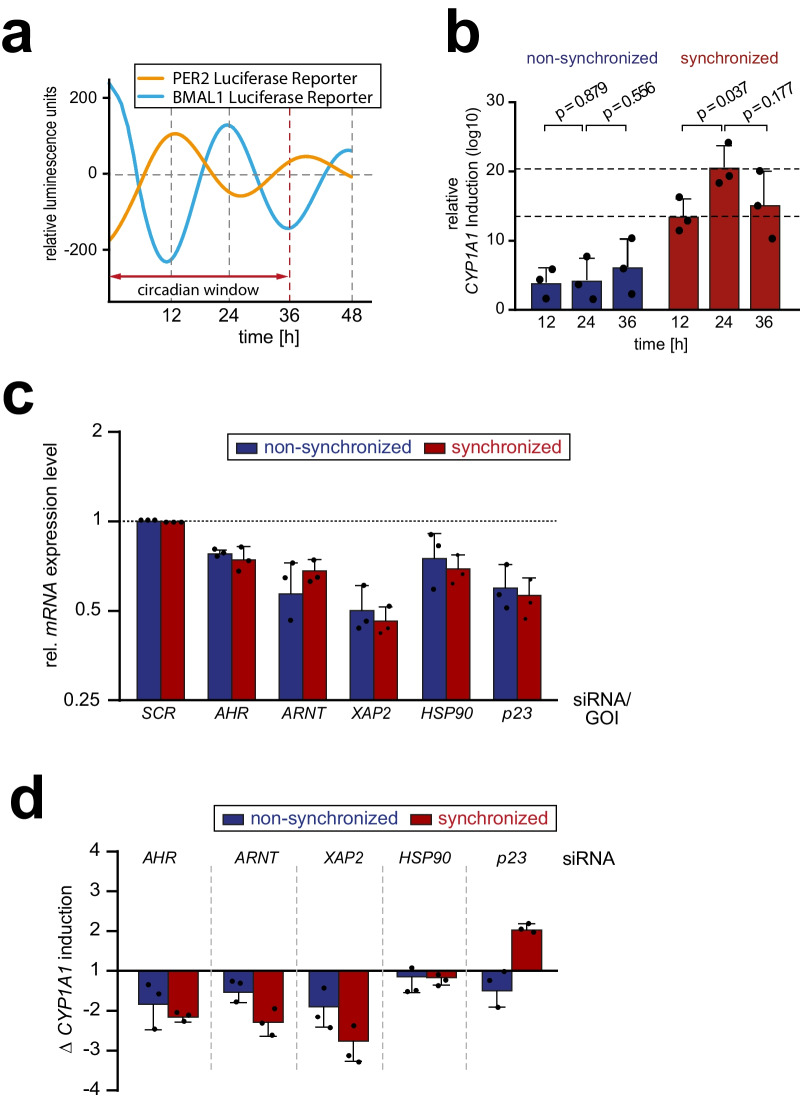
Modulator of the circadian regulated AHR activity.** a**) Representative fitted curve of the circadian rhythm of M13SV1 cells transiently transfected with a *PER2:LUCIFERASE* (orange) or *BMAL1:LUCIFERASE* (blue) circadian reporter plasmid. M13SV1 were transfected via electroporation with the indicated reporter plasmids. 24 h after transfection confluent cells were synchronized by treatment with 1 µM dexamethasone for 1 h and subsequently monitored for 48h. The bioluminescence signal was recorded every 30 min and the obtained measurements was analyzed by the *ChronAlyzer* software. M13SV1 shows a stable synchronization with an anticyclic expression of the *PER2* or *BMAL1* control luciferase **b)** *CYP1A1* induction at different time points in synchronized and non-synchronized M13SV1 cells upon treatment with 0.5 nM TCDD. *CYP1A1* mRNA after TCDD exposure was analyzed by RT-qPCR at indicated timepoints. Fold change was calculated based on the DMSO control of the respective timepoint. In synchronized cells, the TCDD-mediated *CYP1A1* induction displays a circadian pattern peaking at 24 h. Each bar represents the mean ± SD of three independent experiments. The individual data points from the single experiments are displayed. Statistical significance was determined by using an unpaired, one-tailed t-test. **c)** To determine the knock down efficiency of the targeted genes, the mRNA levels of the indicated genes of interest (GOI) were analyzed by RT-qPCR. The knockdown level was calculated by comparing the GOI with the control siRNA transfected cells. Each bar represents the mean ± SD of three independent experiments. The individual data points from the single experiments are displayed. **d)** Decrease and increase of *CYP1A1* induction was determined in M13SV1 cells transfected with siRNA targeting the *AHR* or its co-factors *ARNT*, *XAP2*, *HSP90* and *p23*. The transfected cells were synchronized with 1 µM dexamethasone or left non-synchronized and subsequently exposed to 0.5 nM TCDD for 24 h. *CYP1A1* mRNA levels were determined by RT-qPCR and the change in induction was calculated for the siRNA-targeted gene compared to the control siRNA. Each bar represents the mean ± SD of three independent experiments. The individual data points from the single experiments are displayed.

Except for SP1, none of the AHR co-factors displayed a circadian expression pattern. However, to determine if other co-factors influence the circadian modulation of *CYP1A1* induction, we aimed in the next step to identify modulators of the circadian response to TCDD by siRNA-mediated knock down each co-factor individually. Nevertheless, a salient disadvantage of the HME1 cell system is the substantial difficulty in achieving effective transfection. Consequently, the cell system was changed, and the mechanistic issues were addressed using the M13SV1 human breast epithelial cell line. To ensure a proper circadian synchronization of the cellular system, the M13SV1 cells were transfected with circadian reporter plasmids, and circadian synchrony was monitored over the course of 48 hours (Fig. 3a, S3a). We found that the M13SV1 cells could be effectively synchronized. However, the synchrony was not as stable as in HME1 cells and almost Lost after a 48-hour period. Consequently, this cell system was considered to be suitable for our studies, but only for 36 hours after synchronization (circadian window, Fig. 3a). In order to ascertain whether the M13SV1 cells exhibit a rhythmic response within 36 hours, non-synchronous and synchronous cells were treated with 0.5 nM TCDD and *CYP1A1* induction was determined after 12, 24 and 36 hours (Fig. 3b, S3b). In synchronous cells, a rhythmic *CYP1A1* expression is observed, following the circadian rhythm, with an expression peak at 24 hours of TCDD treatment.

Since the M13SV1 cells were clearly appropriate for our research, we transfected them with siRNAs targeting *AHR* and its co-factors *ARNT*, *XAP2*, *HSP90* and *p23* (Fig. 3b, S3b). Following transfection, the cells were synchronized or left non-synchronized and exposed to DMSO (solvent) or 0.5 nM TCDD for 24 h. The *CYP1A1* induction was analyzed by RT-qPCR determining the mRNA level of *CYP1A1* for the different conditions (Fig. S3c). To identify co-factors affecting *CYP1A1* induction in synchronized cells, the change in induction of control siRNA vs genes of interest (GOI) targeting siRNAs was calculated (Fig. 3c).

As expected, the cells displayed a noticeable induction of *CYP1A1* when treated with TCDD. However, due to siRNA mediated repression of the essential elements of this transcriptional pathway, namely *AHR*, *ARNT* and *XAP2*, the observed induction exhibited a significant decrease.

Notably, comparing the observed effects in synchronized and non-synchronized cells, the reduced induction is more pronounced under synchronous conditions. It is reasonable that these co-factors hold a generic role in this pathway, resulting in a more pronounced reduction when the level of *CYP1A1* mRNA is reduced to an equivalent level in both conditions. The partial depletion of HSP90 had no effect on the induction of CYP1A1.

Considering the established role of p23 as a co-chaperone within this specific pathway, it was hypothesized that its repression would yield a comparable outcome to that of HSP90 on CYP1A1 induction. Surprisingly, the induction of CYP1A1 was found to be twofold higher in cells that had been synchronized and exhibited reduced p23 expression. In contrast, in non-synchronized cells, siRNA-mediated p23 repression resulted in a decrease in *CYP1A1* induction. These results indicate that p23 may act as a distinctive negative regulator of circadian controlled *CYP1A1* induction.

### SP1 and p23 modulate circadian associated AHR activity

Prior experiments revealed two candidate genes that may impact the circadian modulated regulation of *CYP1A1* induction. One promising candidate was the transcription factor SP1, which exhibited a circadian pattern at protein levels peaking at the same time point as *CYP1A1*. The second one was the co-chaperone p23 that uniquely regulated circadian modulated *CYP1A1* induction, although the p23 expression levels were not circadian regulated. To investigate how these two candidate genes modulate *CYP1A1* induction upon TCDD exposure in synchronized cells, we transiently transfected M13SV1 cells with either siRNA targeting *p23* and *SP1* or with a plasmid for ectopic expression of *p23-GFP* and *SP1* (Fig. S4a, S4b). The transfected cells were subsequently synchronized and exposed to increasing doses of TCDD as indicated.

**Fig. 4:**
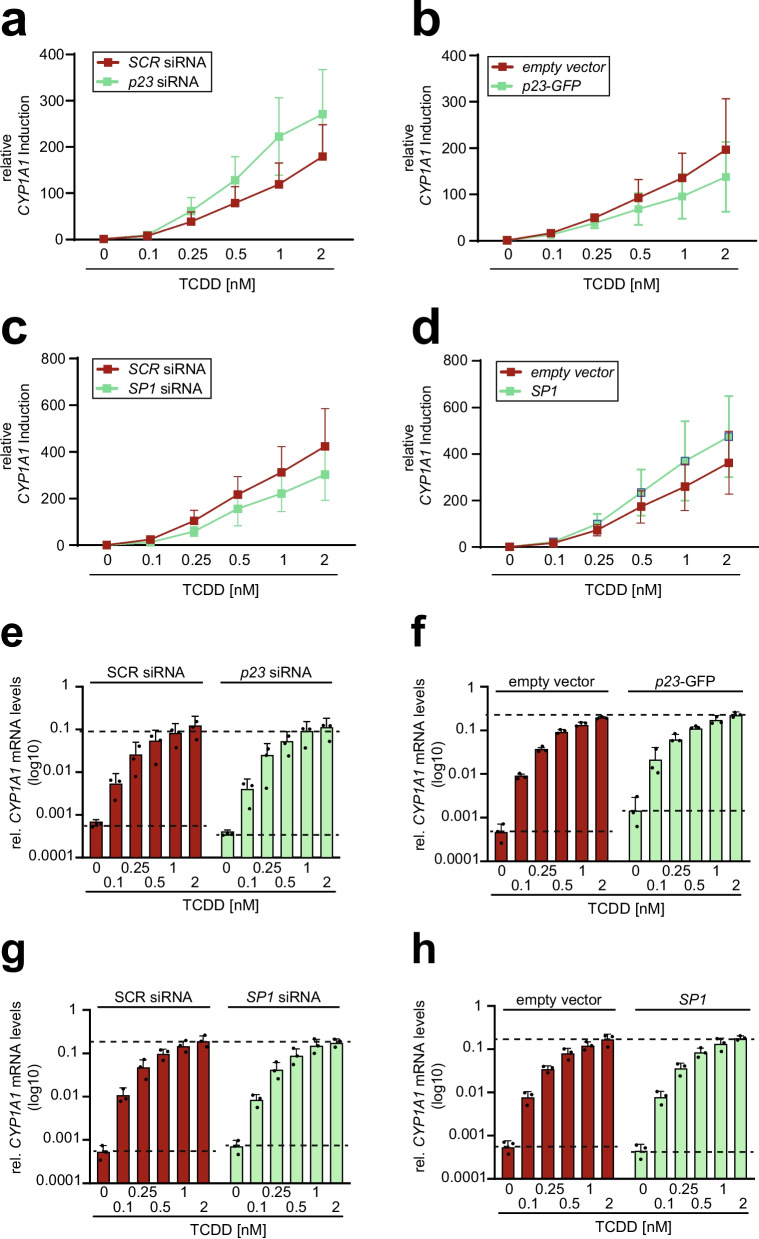
SP1 and p23 modulate circadian associated AHR activity:** a)** Suppression of p23 increases the M13SV1 cell response to TCDD. M13SV1 cells were transiently transfected with scramble (*SCR*) or p23 siRNA. After 48 h of transfection, the cells were synchronized and treated for 24 h with different concentrations of TCDD (0, 0.1, 0.25, 0.5, 1 and 2 nM). The *CYP1A1* mRNA expression was determined by RT-qPCR and the fold change for each TCDD concentration was calculated based on the respective DMSO control. Each point represents the mean ± SD of three independent experiments. **b)** Overexpression of p23 decreases the M13SV1 cell response to TCDD. M13SV1 cells were transfected via electroporation with *empty vector* or *p23-GFP* plasmid. After 48 h of transfection, they were synchronized and treated for 24 h with different concentrations of TCDD (0, 0.1, 0.25, 0.5, 1 and 2 nM). The *CYP1A1* mRNA expression was determined by RT-qPCR and the fold change for each TCDD concentration was calculated based on the respective DMSO control. Each point represents the mean ± SD of three independent experiments. **c)** Suppression of SP1 decreases the M13SV1 cell response to TCDD. M13SV1 cells were transfected with scramble (SCR) or *SP1* siRNA. After 48 h of transfection, they were synchronized and treated for 24 h with different concentrations of TCDD (0, 0.1, 0.25, 0.5, 1 and 2 nM). The *CYP1A1* mRNA expression was determined by RT-qPCR and the fold change for each TCDD concentration was calculated based on the respective DMSO control. Each point represents the mean ± SD of three independent experiments. **d)** Overexpression of SP1 enhances the M13SV1 cell response to TCDD. M13SV1 cells were transfected via electroporation with empty vector or SP1 overexpression plasmid. After 48 h of transfection, they were synchronized and treated for 24 h with different concentrations of TCDD (0, 0.1, 0.25, 0.5, 1 and 2 nM). The *CYP1A1* mRNA expression was determined by RT-qPCR and the fold change for each TCDD concentration was calculated based on the respective DMSO control. Each point represents the mean ± SD of three independent experiments. **e)** Relative *CYP1A1* mRNA expression in TCDD treated synchronized cells transfected with *SCR* or *p23* siRNA. 48 h after transfection, M13SV1 cells were synchronized with dexamethasone (1 µM) for 1 h and subsequently treated with different TCDD concentration (0, 0.1, 0.25, 0.5, 1 and 2 nM) for 24 h. The mRNA levels of *CYP1A1* were analyzed by RT-qPCR and normalized to the endogenous *B2M*. Each bar represents the mean ± SD of three independent experiments. The individual data points from the single experiments are displayed. **f)** Relative *CYP1A1* mRNA expression in TCDD treated synchronized cells transfected with *empty vector* or *p23-GFP* plasmid DNA. 48 h after transfection, M13SV1 cells were synchronized with dexamethasone (1 µM) for 1 h and subsequently treated with different TCDD concentration (0, 0.1, 0.25, 0.5, 1 and 2 nM) for 24 h. The mRNA levels of *CYP1A1* were analyzed by RT-qPCR and normalized to the endogenous *B2M*. Each bar represents the mean ± SD of three independent experiments. The individual data points from the single experiments are displayed. **g)** Relative *CYP1A1* mRNA expression in TCDD treated synchronized cells transfected with *SCR* or *SP1* siRNA. 48 h after transfection, M13SV1 cells were synchronized with dexamethasone (1 µM) for 1 h and subsequently treated with different TCDD concentration (0, 0.1, 0.25, 0.5, 1 and 2 nM) for 24 h. The mRNA levels of *CYP1A1* were analyzed by RT-qPCR and normalized to the endogenous *B2M*. Each bar represents the mean ± SD of three independent experiments. The individual data points from the single experiments are displayed. **h)** Relative *CYP1A1* mRNA expression in TCDD treated synchronized cells transfected with *empty vector* or *SP1* plasmid DNA. 48 h after transfection, M13SV1 cells were synchronized with dexamethasone (1 µM) for 1 h and subsequently treated with different TCDD concentration (0, 0.1, 0.25, 0.5, 1 and 2 nM) for 24 h. The mRNA levels of *CYP1A1* were analyzed by RT-qPCR and normalized to the endogenous *B2M*. Each bar represents the mean ± SD of three independent experiments. The individual data points from the single experiments are displayed

Remarkably, not only the reduction of *p23* expression by siRNA knock down resulted in an enhanced stimulation of *CYP1A1* expression upon TCDD treatment in a dose-dependent manner (Fig. 4a). *Vice versa*, the overexpression of p23 by transfection with a p23 expression vector led to a diminished dose response (Fig. 4b). Interestingly, our results for SP1 were reversed. The repression of SP1 led to a reduced dose response (Fig. 4c), while overexpression resulted in an increased dose response (Fig. 4d). A detailed analysis of mRNA expression revealed that the dynamic range of cellular response is modulated by alterations in *p23* expression. A repression of *p23* expended the dynamic response range whereas the overexpression of *p23* reduced the dynamic response range (Fig. 4e and f). Interestingly, we demonstrated in the earlier study, that circadian synchronized cells exhibit a higher dynamic response range in comparison to non-synchronized cells (Ndikung et al. 2020). The role of SP1 in the circadian modulation of *CYP1A1* appears to be similar, albeit with a significantly lower effect level (Fig. 4g and h). These findings suggest that SP1 and p23 are counter-regulators of circadian-driven *CYP1A1* induction upon exposure to AHR ligands, such as TCDD.

To ensure that the repression or overexpression of *SP1* and *p23* did not interfere with the circadian synchrony *per se*, we transfected the circadian reporter cells, *hTERT*-HME1-*PER2,* with the candidates and monitored the circadian rhythm for 84 h. As both candidates have not been described as circadian regulators before, we did not expect any impairment of circadian synchrony. Indeed, the analysis of the bioluminescent signal of the transiently transfected reporter plasmids confirmed that there were no changes in the circadian rhythm following the repression or overexpression of *SP1* and *p23* (Fig. S4c).

Finally, to exclude the possibility that the repression or overexpression of *p23* or *SP1* interferes *per se* with the dose-dependent *CYP1A1* induction, non-synchronous cells were treated with 0.5 nM and 2 nM TCDD for 24 h, and the *CYP1A1* mRNA induction was determined by qPCR. In contrast to the synchronous cell, no significant alterations in *CYP1A1* expression were observed due to suppression or overexpression of *SP1* and *p23* (Fig. S4d).

### AHR interaction with the clock core complex BMAL1/CLOCK

In our previous experiments, we demonstrated the circadian modulated regulation of AHR target gene induction and revealed SP1 and p23 as central regulators of the circadian-driven transcriptional activity of AHR upon TCDD treatment. However, a direct link of the AHR pathway and circadian machinery is still missing, since AHR and its major co-factors seem not to be direct clock control genes in human breast cells. Thus, we hypothesized that the regulation is achieved by a protein-protein interaction between the clock machinery and the AHR.

**Fig. 5:**
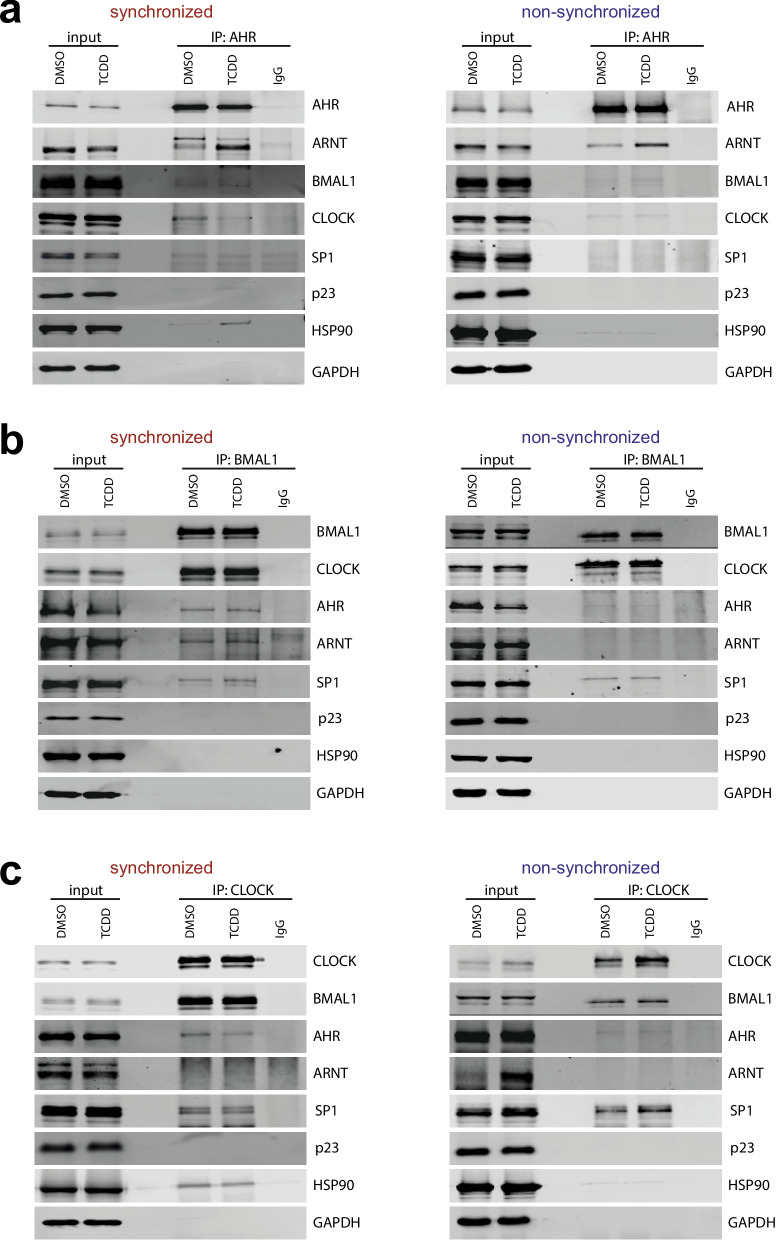
The clock core complex BMAL1/CLOCK directly interacts with the AHR/ARNT complex. HME1 cells were synchronized with 1 µM dexamethasone for 1 h and after 23 h cultivation exposed to 2 nM TCDD for additional 1 h. **a)** Immunoprecipitation of endogenous AHR. AHR was immunoprecipitated from whole-cell lysates and AHR, ARNT, BMAL1, CLOCK, SP1, p23, HSP90 and GAPDH (as control) were detected on Western blots. Representative Western blots are shown from independent experiments. **b)** Immunoprecipitation of endogenous BMAL1. BMAL1 was immunoprecipitated from whole-cell lysates and BMAL1, CLOCK, AHR, ARNT, SP1, p23, HSP90 and GAPDH (as control) were detected on Western blots. **c)** Immunoprecipitation of endogenous CLOCK. CLOCK was immunoprecipitated from whole-cell lysates and CLOCK, BMAL1, AHR, ARNT, SP1, HSP90 and GAPDH (as control) were detected on Western blots. Representative Western blots are shown from independent experiments. For all interaction studies, an antibody mock control was included to determine unspecific bands from the antibody solution.

In order to address this, interaction studies were conducted by immunoprecipitating endogenous AHR of circadian synchronized HME1 cells treated with DMSO or TCDD and comparing it with non-synchronous cells also treated with DMSO and TCDD. In detail, HME1 cells were synchronized with 1 µM dexamethasone for 1 h or left non-synchronized, cultivated for 23 h and subsequently exposed to 2 nM TCDD for additional 1 h. This sequential treatment scenario ensures optimal conditions for interaction studies that favor the detection of directly induced complexes and minimize possible secondary, compensatory effects as well as steady-state formation that may occurs in long-term treated cells. The interaction partners of AHR were identified and analyzed by Western blotting. As expected, a clear increased interaction between AHR and ARNT could be observed in TCDD-treated cells (Fig. 5a). This finding served as a positive control for synchronized and non-synchronized conditions to ensure that the precipitated AHR complex was active and reflected the expected results. The observation of p23 not binding to AHR, even under the control conditions, is not unexpected, given that p23 is only bound to HSP90 in the cytosolic complex. In the case of HSP90, a strong interaction could not be observed under all conditions. However, the TCDD-treated synchronized cells showed a modest increase in the interaction of AHR and HSP90.

Interestingly, we identified a very weak interaction between AHR and the core clock protein BMAL1 and its primary partner CLOCK in synchronized cells.

Nevertheless, the interaction between BMAL1/CLOCK does not appear to be particularly strong, and in the case of BMAL1, does not depend on the treatment with TCDD. The situation is different with CLOCK, where the interaction seems to disappear upon TCDD treatment. It is important to note that the interactions in the non-synchronized cells are even weaker than those in the synchronized cells. In particular for CLOCK, we could not find a stronger interaction with AHR when treated with DMSO.

In order to conduct a more profound analysis of the interaction complex and to verify the findings of the AHR interaction study, a reverse study was conducted with BMAL1. To this end, HME1 cells were synchronized or left non-synchronized and treated as previously described. Subsequently, an immunoprecipitation of the endogenous BMAL1 was performed. As interaction control for an active complex, we detected CLOCK, the major co-factor of BMAL1 (Fig. 5b). Indeed, AHR and ARNT were clearly identified in this complex in circadian synchronized cells, however, with no interaction difference, particular with ARNT, between DMSO and TCDD treated cells. It is important to note that this interaction between BMAL1 and AHR/ARNT is not detectable in non-synchronized cells. Interestingly, in this setup we could also identify SP1 as a player in this network, while no interaction with p23 or HSP90 could be detected.

To further elucidate the composition of the circadian complex, we finally conducted a CLOCK immunoprecipitation and confirmed the presence of the relevant players (Fig. 5c). The interaction of major co-factor BMAL1 served again as control for an active circadian complex. Furthermore, a clear interaction was observed between CLOCK and AHR, as well as CLOCK and SP1, while no interaction was identified with ARNT. In accordance with the findings of previous interaction studies of AHR and BMAL1, the interaction of CLOCK with AHR and ARNT was found to be significantly weaker in non-synchronized cells. As previously noted, no TCDD-dependent change was observed in the composition of these complexes. It is noteworthy that we observe a clear interaction of CLOCK with HSP90 in synchronized cells.

Taken together, these findings indicate a potential interaction between an active circadian BMAL1/CLOCK complex and the AHR/ARNT complex, particularly in synchronized cells. This interaction could function as a direct connection between the clock machinery and the AHR pathway. The BMAL1/CLOCK core complex appears to function as a molecular bridge between the circadian-regulated SP1 transcription factor and the AHR/ARNT response complex, thereby maintaining the circadian expression of target genes such as CYP1A1.

## Discussion

The determination of the toxicity of chemicals is of the utmost importance in toxicological assessments, particularly in the context of dioxins, which are the subject of ongoing scientific and public health interest. Dioxins are primarily the result of industrial processes, although they can also be generated as a consequence of natural processes, including forest fires and volcanic eruptions. Moreover, dioxins are considered to be among the most persistent environmental contaminants, resulting in widespread exposure to humans across the globe. The primary route of exposure is through the food chain, where they tend to accumulate in the tissues of higher organisms. Dioxins are highly toxic, and exposure can result in a range of adverse health outcomes, including reproductive and developmental issues and cancer.

These assessments are typically derived from the findings of animal experiments and epidemiological studies. However, epidemiological studies are only available to a limited extent, and animal testing are considered obsolete in the context of next-generation risk assessment. This necessitates the development of advanced *in vitro* test methods and experimental designs that most closely align with the realistic scenario of exposure, including Questions of dose response. To this end, the primary objective of current research is the reconstruction of tissue 3D structures, which is being employed in the development of advanced *in vitro* systems. Despite all the complexity of such systems, they still have wide range of limitations, including the absence of inherent key regulatory mechanisms (Habanjar et al. 2021).

In particular, the impact of the circadian rhythm on the outcomes of *in vitro* toxicological studies has been insufficiently explored. The circadian rhythm is a fundamental biological mechanism that regulates and modulates a multitude of physiological processes in the human body, even at the molecular level of a single cell. In particular, the circadian rhythm plays a pivotal role in maintaining the metabolic response necessary for the proper functioning of tissues and organs, and thus for the maintenance of overall health. This regulation includes also the cellular response to potentially harmful substances or xenobiotics. A number of studies, both *in vivo* and *in vitro*, have described a circadian response to chemicals, with a wide range of modes of action (Dallmann et al. 2016; Sancar and Van Gelder 2021; Zheng et al. 2021). Indeed, also our previous research demonstrated that synchronizing cells *in vitro* markedly enhances the biological response to TCDD, as evidenced by the increased induction of *CYP1A1* by AHR signaling. To emphasize the significance of this observation, it is crucial to elucidate the underlying molecular mechanisms.

This study links the circadian rhythm to a highly relevant toxicological pathway in human breast cells and provides the first insight into circadian regulation at the molecular level. In particular, we demonstrate a direct interaction of the xenobiotic response complex AHR/ARNT with the circadian BMAL1/CLOCK complex in circadian synchronized human breast epithelial cells. This interaction may trigger a circadian activity of the AHR/ARNT complex, which subsequently induces, under the same conditions, its target gene in a circadian manner. Based on this finding we postulate, for the first time, a circadian regulation of the active AHR/ARNT complex by the BMAL1/CLOCK partner complex. We suggest that clock components not only act as independent transcription factors regulating their own targets, but can also influence the activation of other protein complexes. However, the molecular nature of these interactions is still unclear and requires further study.

Moreover, we could identify two key proteins modulating the circadian regulation of AHR responses. The versatile transcription factor SP1 acts as an enhancer of the circadian regulated response and, more strikingly, the co-chaperon p23 appears to be a negative regulator of the circadian response of AHR/ARNT activity. SP1 has been described as a co-activator of AHR transcriptional activity (Kobayashi et al. 1996) and has been shown to positively regulate BMAL1 transcription (Xiao et al. 2013). Interestingly, we could also connect SP1 expression to the circadian machinery, since we identified a circadian expression pattern of SP1 protein levels with a peak at 24/48 h and Low expression at 12/36 h after synchronization, which is aligned with the circadian expression pattern of *CYP1A1* induction. Furthermore, our findings revealed a direct interaction between SP1 and the BMAL1/CLOCK complex, but not with AHR/ARNT. While BMAL1/CLOCK directly interacts with AHR, it may serve as a molecular bridge between circadian-regulated SP1 and AHR. It is conceivable that SP1 and BMAL1/CLOCK promote a circadian-regulated localization of AHR next to the XRE at the promoter, where it can interact with ARNT and trigger the rhythmic *CYP1A1* induction.

Nevertheless, additional research is needed to determine whether SP1 is subject to translational or post-translational circadian regulation. For instance, it would be of interest to determine whether the protein stability is regulated in a circadian manner. In this context, a study by Gong et al. (2014) has linked the stability of the SP1 protein to its regulation by sumoylation. Furthermore, sumoylation is known to regulate the stability of clock genes, such as Per2 or BMAL1 (Cardone et al. 2005; Chen et al. 2021), and could therefore also be relevant for the circadian oscillation of the SP1 protein levels.

The result that repression of p23 increased the transcriptional activity of AHR/ARNT exclusively in circadian-synchronized cells emerged as a more surprising finding. In contrast to SP1, the co-chaperone p23 does not appear to be regulated in a circadian manner in human breast cells, although, a circadian expression profile was reported in rat liver (Almon et al. 2008). The observation remarkably shows that a circadian effect does not always have to be caused by circadian regulated genes. Specifically, the chaperone p23 appears to enhance circadian effects.

This study offers preliminary insights into the emerging field of circadian aspects in toxicological response and its implications for health protection. In this regard, a primary concern is the disruption of the circadian rhythm and its association with the development of various diseases (Fishbein et al. 2021; Zheng et al. 2021; Mihelakis et al. 2022). An understanding of the modulation of cellular responses by the circadian rhythm may provide insight into the potential initiation or promotion of disease pathology by circadian distribution. Recent studies report that common diseases such as metabolic syndrome, cardiovascular diseases, and cancer are highly associated with circadian disruption (Fishbein et al. 2021). In the field of chronopharmacology, understanding the mechanism behind the circadian regulation of pathways would help to identify the most optimal treatment window for time-dependent drug administration (Dobrek 2021; Mihelakis et al. 2022).

In addition to pharmacological approaches, there has been growing interest in the field of regulatory toxicology in understanding the molecular basis of toxicological responses. It is therefore of great importance to gain a comprehensive understanding of regulatory processes, especially in the context of the circadian framework. To understand and test the toxicity of chemicals and drugs, the adverse outcome pathway (AOP) concept has proven to be a powerful tool for generating knowledge and developing assays to identify health risks. Within an AOP, single key events (KE) are captured that mediate adversity, covering their relationship and regulation. This will provide a deeper understanding of the toxic effects of chemicals, which subsequently allows for better risk assessments strategies. While the identification of KEs is relatively straightforward, the overall understanding of the regulatory network and in particular (quantitative) KE-KE relationships is a much more complex issue. With this study we provide new evidence that the circadian machinery has a significant impact on the AHR regulation network, which might help to improve AHR-related AOPs. Currently, there are 10 AOPs being considered that link AHR activation to diseases such as breast and liver cancer (https://aopwiki.org). Interestingly, the disruption of the circadian rhythm, for example by shift work, is strongly associated with an increased incidence of breast cancer (Gehlert et al. 2020). Our findings link the AHR pathway directly with the circadian machinery, potentially improving AHR-related AOPs and enhancing the understanding of the associated disease pathology and development.

In conclusion, we identify first insights into the complex nature of circadian driven regulation of AHR function in human cells, in particular in human breast epithelia non-tumor cells. These results highlight the significance of understanding the physiological regulation of components that are involved in crucial metabolic processes, such as xenobiotic metabolism. This enables a better understanding of the toxicological processes relevant to human health risk assessment and opens up the possibility of developing *in vitro* systems with greater biological relevance.

## Supplementary Information

Below is the link to the electronic supplementary material.

ESM 1 **Figure 1:** **a)** Representative bioluminescence recordings of non-synchronized (blue) and synchronized (red) circadian HME1 reporter cells expressing luciferase under control of the human *PER2* promoter.**b)** Relative *CYP1A1* mRNA expression (ΔCt) in non-synchronized and synchronized HME1 cells treated with DMSO (control) or TCDD (0.5 nM) for 12, 24, 36 and 48 h. The mRNA expression was determined by RT-PCR and normalized to the endogenous control *B2M*. Each data point represents the mean ± SD of three independent experiments. **c)** Relative *de novo* *CYP1A1* mRNA expression (ΔCt) in non-synchronized and synchronized HME1 cells treated with DMSO (control) or TCDD for 12, 24, 36 and 48 h. The mRNA of the nucleus was used for the newly synthesized (*de novo*) *CYP1A1* mRNA expression. The analysis was conducted by RT-qPCR and normalized to the endogenous control *B2M*. Each data point represents the mean ± SD of three independent experiments. (PNG 341 KB)

Supplementary file1 (EPS 1833 KB)

ESM 2 **Figure 2: a)** Relative *AHR*, *ARNT*, *XAP2*, *SP1*, *HSP90*, *p23,* and* AHRR* mRNA expression (ΔCt) in non-synchronized and synchronized HME1 cells treated with DMSO (control) or TCDD (0.5 nM) for 12, 24, 36 and 48 h. The mRNA expression was determined by RT-qPCR and normalized to the endogenous control *B2M*. Each data point represents the mean ± SD of three independent experiments. (PNG 503 KB)

Supplementary file2 (EPS 1997 KB)

ESM 3 **Figure 3: a) **Representative bioluminescence measurements of synchronized M13SV1 cells transiently transfected with a *PER2:LUCIFERASE* (orange) or *BMAL1:LUCIFERASE* (blue) circadian reporter plasmid. M13SV1 were transfected via electroporation with the *PER2* and *BMAL1* reporter plasmids and 24 h after transfection synchronized with 1 µM dexamethasone for 1 h and subsequently monitored for 48 h. The bioluminescence signal was recorded every 30 min. **b)** Relative *CYP1A1* mRNA expression (ΔCt) in non-synchronized and synchronized M13SV1 cells treated with 0.5 nM TCDD at indicated timepoints. The mRNA expression was determined by RT-PCR and normalized to the endogenous control *B2M*. Each data point represents the mean ± SD of three independent experiments. **c)** The knockdown efficiency of the targeted genes was determined by RT-qPCR analysis for the mRNA levels of the indicated genes of interest (GOI). The analysis was conducted by RT-qPCR and normalized to the endogenous control *B2M*. Each bar represents the mean ± SD of three independent experiments. **d)** Alterations of *CYP1A1* induction were determined in M13SV1 cells transfected with siRNA targeting the *AHR* or its co-factors *ARNT*, *XAP2*, *HSP90* and *p23*. The transfected cells were synchronized with 1 µM dexamethasone or left non-synchronized and subsequently exposed to 0.5 nM TCDD for 24 h. The mRNA levels were determined by RT-qPCR and normalized to the endogenous control *B2M*. The *CYP1A1* induction was calculated by comparing the TCDD treated cells with the DMSO control treated cells. Each bar represents the mean ± SD of three independent experiments. (PNG 816 KB)

Supplementary file3 (EPS 2135 KB)

ESM 4 **Figure 4: a)** The efficiency of *p23* siRNA and *p23-GFP* overexpression plasmid to achieve a p23 downregulation or upregulation, respectively, was calculated by analyzing the *p23* mRNA levels via RT-qPCR. The knockdown or overexpression level was calculated by comparing the p23 expression of p23 downregulated or upregulated cells with the p23 expression of control (SCR or empty vector) transfected cells. Similarly, the downregulation efficiency of *SP1* siRNA was determined. Each bar represents the mean ± SD of three independent experiments.**b)** Representative Western blots of M13SV1 cells transfected with *SP1* and *p23* siRNA or with *p23-GFP* and *SP1* overexpression plasmid. The p23 or SP1 repression or overexpression efficiency was determined at protein level by detecting p23 and SP1 on the Western blots. β-Actin served as loading control. **c)** Representative bioluminescence measurements of synchronized HME1 cells expressing *PER2:LUCIFERASE* circadian reporter plasmid (HME1_PLB cells). HME1_PLB cells were transfected via electroporation with *empty vector*,*p23-GFP* overexpression plasmid, *p23* silencing plasmid, *SP1* overexpression plasmid and *SP1* siRNA. 24 h after transfection, the cells were synchronized with 1 µM dexamethasone for 1 h and subsequently monitored for 48h. The bioluminescence signal was recorded every 30 min. The fitted bioluminescence curves are shown on the top two graphs and the raw bioluminescence measurements at the bottom two graphs. **d)**. M13SV1 cells were transiently transfected with siRNA (targeting scramble (*SCR*), *p23* or *SP1*) or plasmid DNA (ev, SP1, p23-GFP). After 48 h of transfection, the cells were treated for 24 h with different concentrations of TCDD (0, 0.5, and 2 nM). The *CYP1A1* mRNA expression was determined by RT-qPCR and the fold change for each TCDD concentration was calculated based on the respective DMSO control. Each point represents the mean ± SD of three independent experiments. (PNG 730 KB)

Supplementary file4 (EPS 2336 KB)

## Data Availability

No datasets were generated or analysed during the current study.
