# Supplementary figures and images for: SP1 and p23 play a crucial role in the circadian target gene induction of activated aryl hydrocarbon receptor in human breast cells
Source: Cell Biol Toxicol. 2025 Sep 12;41(1):130. doi: 10.1007/s10565-025-10080-0 (PMC12426158; doi:10.1007/s10565-025-10080-0)

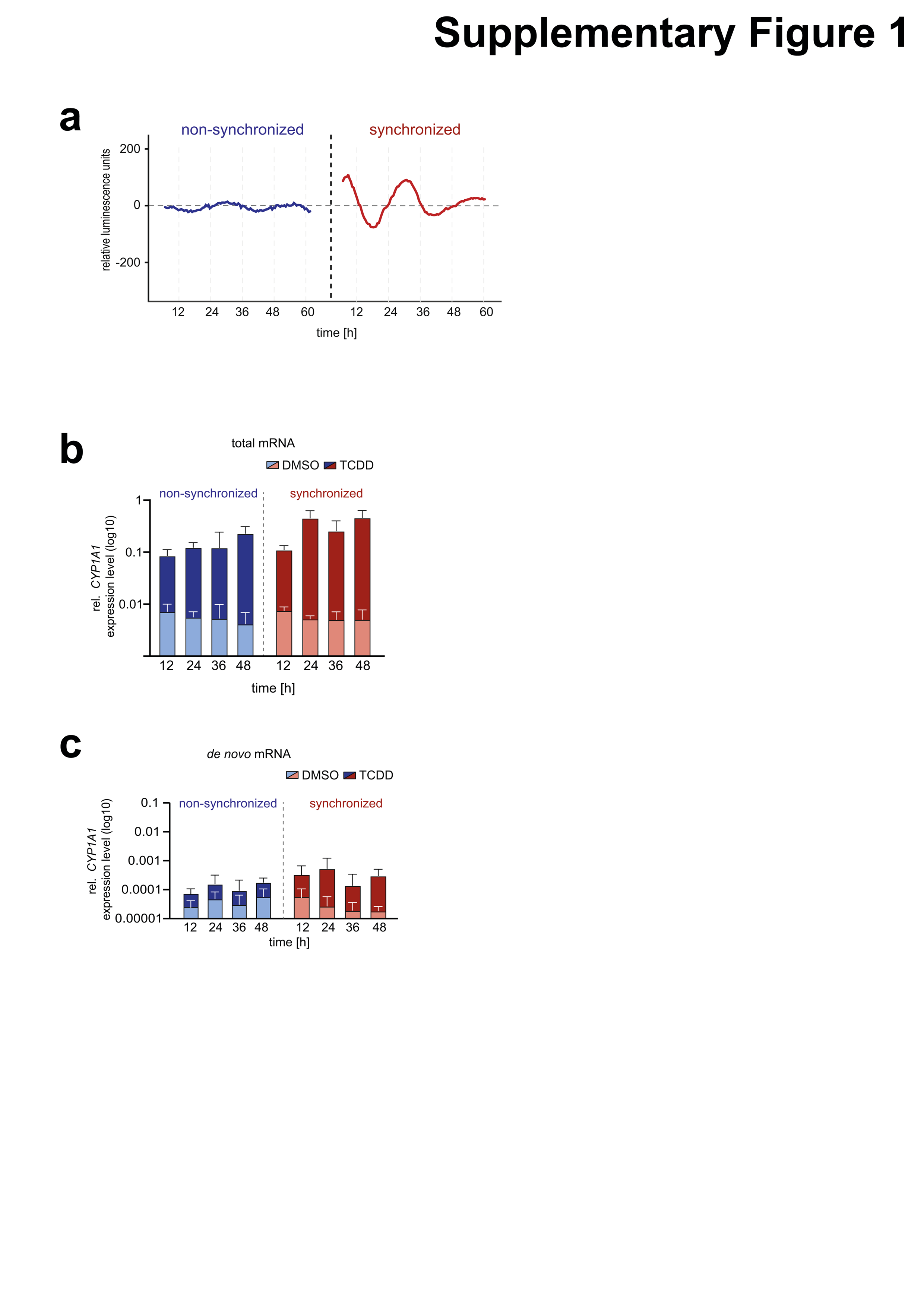

Supplement: Supplementary file 1 — Figure 1: a) Representative bioluminescence recordings of non-synchronized (blue) and synchronized (red) circadian HME1 reporter cells expressing luciferase under control of the human PER2 promoter.b) Relative CYP1A1 mRNA expression (ΔCt) in non-synchronized and synchronized HME1 cells treated with DMSO (control) or TCDD (0.5 nM) for 12, 24, 36 and 48 h. The mRNA expression was determined by RT-PCR and normalized to the endogenous control B2M. Each data point represents the mean ± SD of three independent experiments. c) Relative de novo CYP1A1 mRNA expression (ΔCt) in non-synchronized and synchronized HME1 cells treated with DMSO (control) or TCDD for 12, 24, 36 and 48 h. The mRNA of the nucleus was used for the newly synthesized (de novo) CYP1A1 mRNA expression. The analysis was conducted by RT-qPCR and normalized to the endogenous control B2M. Each data point represents the mean ± SD of three independent experiments. (PNG 341 KB) [file 10565_2025_10080_Fig6_ESM.png]

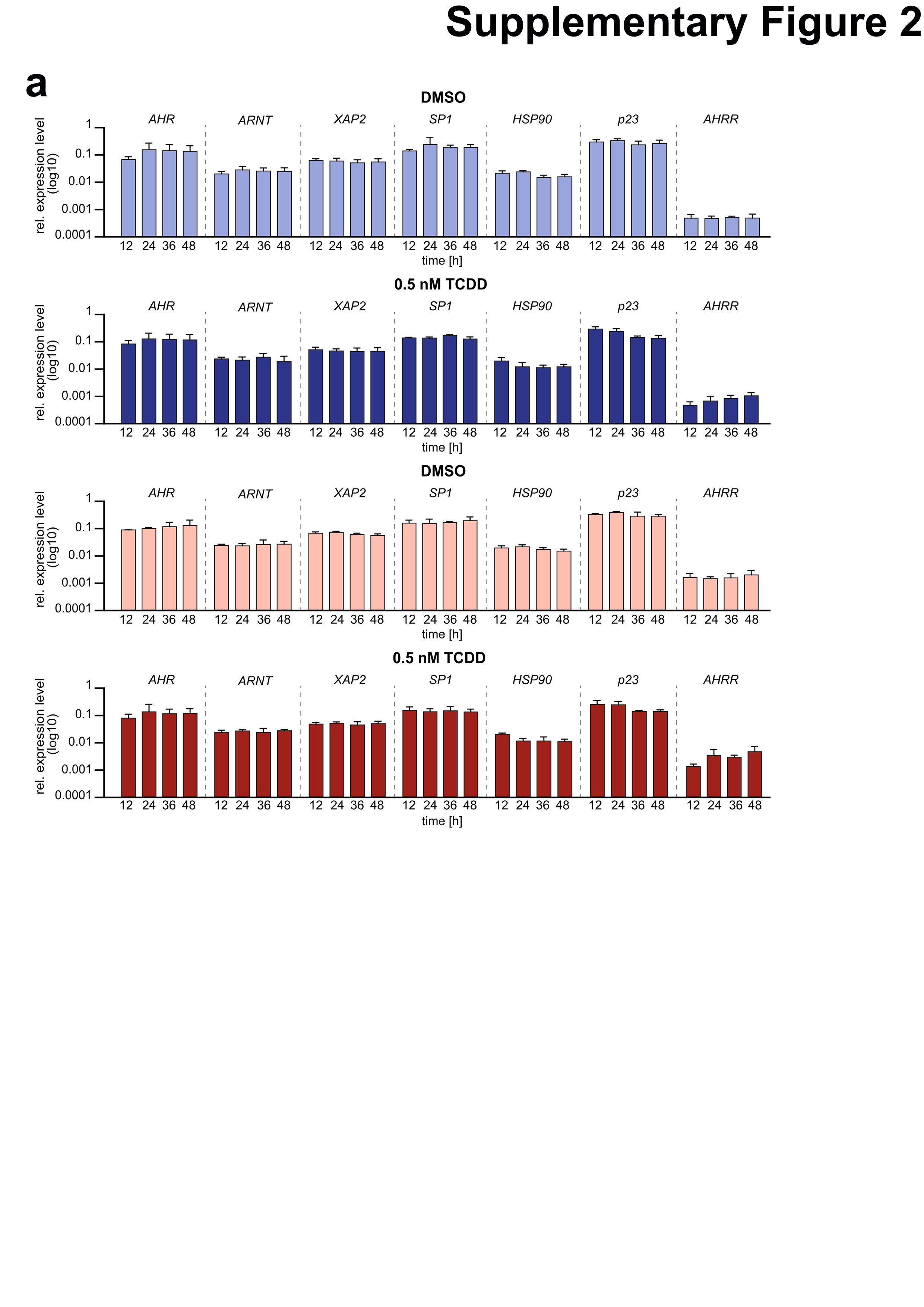

Supplement: Supplementary file 3 — Figure 2: a) Relative AHR, ARNT, XAP2, SP1, HSP90, p23, and AHRR mRNA expression (ΔCt) in non-synchronized and synchronized HME1 cells treated with DMSO (control) or TCDD (0.5 nM) for 12, 24, 36 and 48 h. The mRNA expression was determined by RT-qPCR and normalized to the endogenous control B2M. Each data point represents the mean ± SD of three independent experiments. (PNG 503 KB) [file 10565_2025_10080_Fig7_ESM.png]

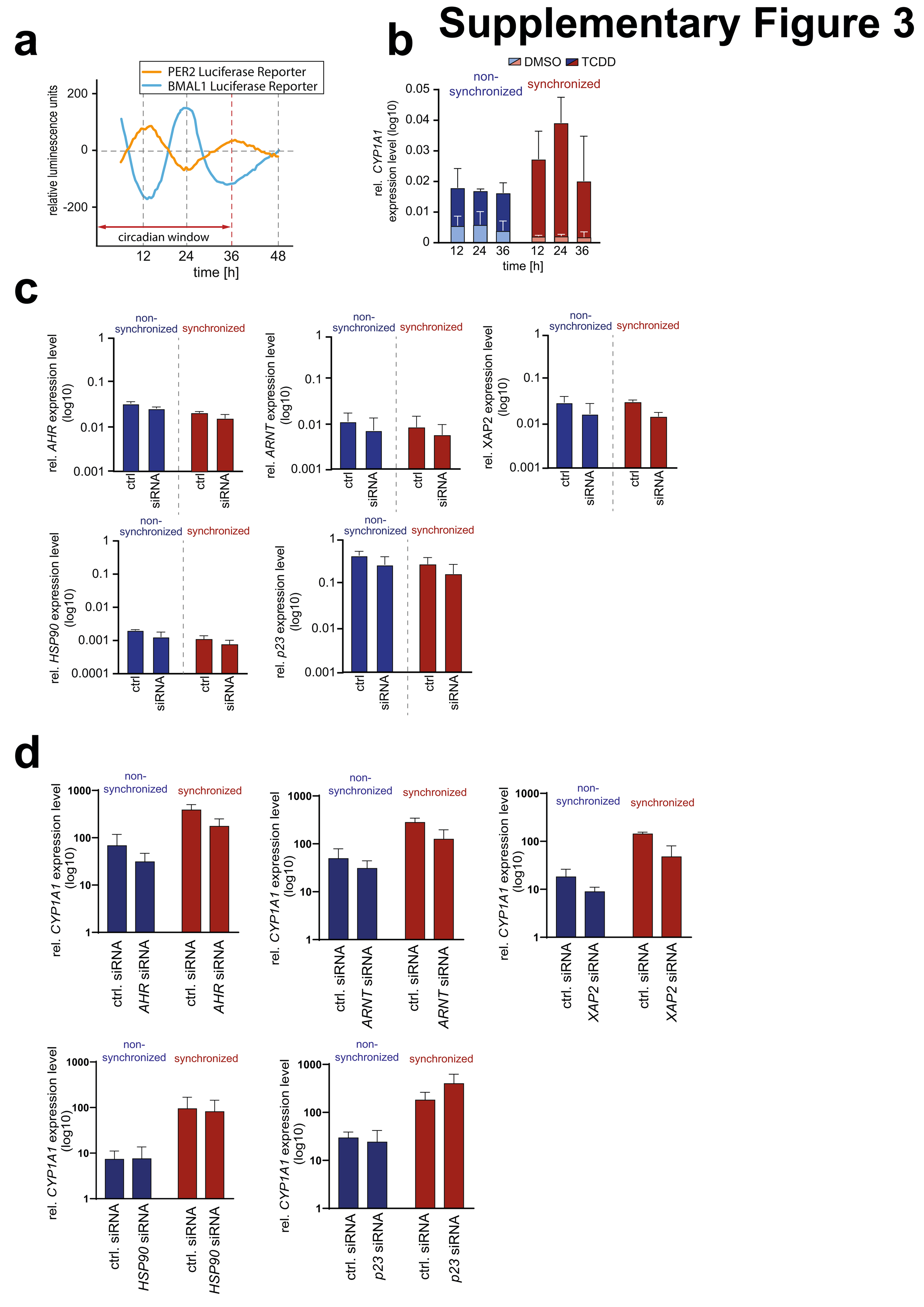

Supplement: Supplementary file 5 — Figure 3: a) Representative bioluminescence measurements of synchronized M13SV1 cells transiently transfected with a PER2:LUCIFERASE (orange) or BMAL1:LUCIFERASE (blue) circadian reporter plasmid. M13SV1 were transfected via electroporation with the PER2 and BMAL1 reporter plasmids and 24 h after transfection synchronized with 1 µM dexamethasone for 1 h and subsequently monitored for 48 h. The bioluminescence signal was recorded every 30 min. b) Relative CYP1A1 mRNA expression (ΔCt) in non-synchronized and synchronized M13SV1 cells treated with 0.5 nM TCDD at indicated timepoints. The mRNA expression was determined by RT-PCR and normalized to the endogenous control B2M. Each data point represents the mean ± SD of three independent experiments. c) The knockdown efficiency of the targeted genes was determined by RT-qPCR analysis for the mRNA levels of the indicated genes of interest (GOI). The analysis was conducted by RT-qPCR and normalized to the endogenous control B2M. Each bar represents the mean ± SD of three independent experiments. d) Alterations of CYP1A1 induction were determined in M13SV1 cells transfected with siRNA targeting the AHR or its co-factors ARNT, XAP2, HSP90 and p23. The transfected cells were synchronized with 1 µM dexamethasone or left non-synchronized and subsequently exposed to 0.5 nM TCDD for 24 h. The mRNA levels were determined by RT-qPCR and normalized to the endogenous control B2M. The CYP1A1 induction was calculated by comparing the TCDD treated cells with the DMSO control treated cells. Each bar represents the mean ± SD of three independent experiments. (PNG 816 KB) [file 10565_2025_10080_Fig8_ESM.png]

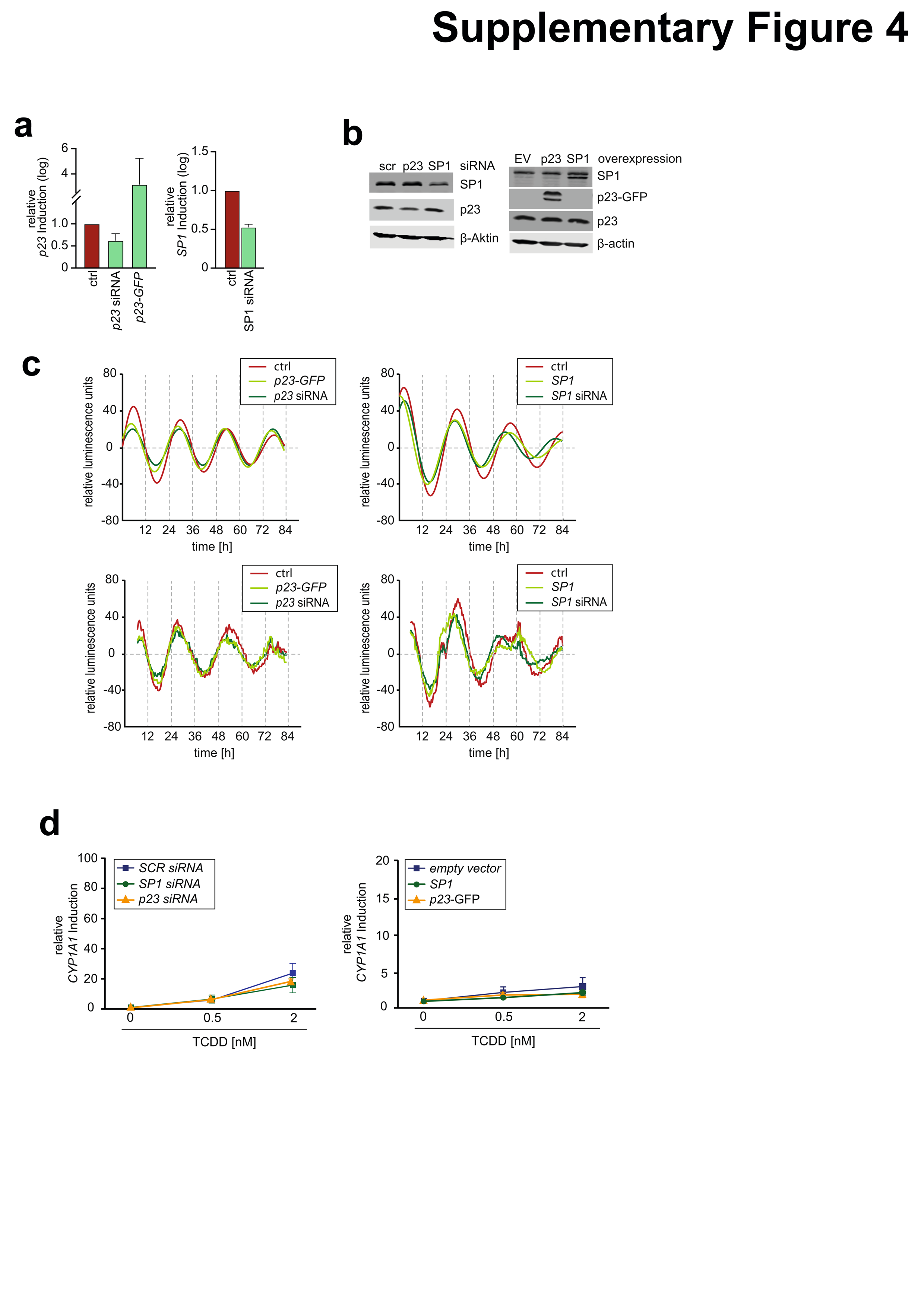

Supplement: Supplementary file 7 — Figure 4: a) The efficiency of p23 siRNA and p23-GFP overexpression plasmid to achieve a p23 downregulation or upregulation, respectively, was calculated by analyzing the p23 mRNA levels via RT-qPCR. The knockdown or overexpression level was calculated by comparing the p23 expression of p23 downregulated or upregulated cells with the p23 expression of control (SCR or empty vector) transfected cells. Similarly, the downregulation efficiency of SP1 siRNA was determined. Each bar represents the mean ± SD of three independent experiments.b) Representative Western blots of M13SV1 cells transfected with SP1 and p23 siRNA or with p23-GFP and SP1 overexpression plasmid. The p23 or SP1 repression or overexpression efficiency was determined at protein level by detecting p23 and SP1 on the Western blots. β-Actin served as loading control. c) Representative bioluminescence measurements of synchronized HME1 cells expressing PER2:LUCIFERASE circadian reporter plasmid (HME1_PLB cells). HME1_PLB cells were transfected via electroporation with empty vector,p23-GFP overexpression plasmid, p23 silencing plasmid, SP1 overexpression plasmid and SP1 siRNA. 24 h after transfection, the cells were synchronized with 1 µM dexamethasone for 1 h and subsequently monitored for 48h. The bioluminescence signal was recorded every 30 min. The fitted bioluminescence curves are shown on the top two graphs and the raw bioluminescence measurements at the bottom two graphs. d). M13SV1 cells were transiently transfected with siRNA (targeting scramble (SCR), p23 or SP1) or plasmid DNA (ev, SP1, p23-GFP). After 48 h of transfection, the cells were treated for 24 h with different concentrations of TCDD (0, 0.5, and 2 nM). The CYP1A1 mRNA expression was determined by RT-qPCR and the fold change for each TCDD concentration was calculated based on the respective DMSO control. Each point represents the mean ± SD of three independent experiments. (PNG 730 KB) [file 10565_2025_10080_Fig9_ESM.png]
